# Unmet needs in allergic rhinitis: international survey on management of allergic rhinitis by physician and patient. The physicians' view (ISMAR 2 study)

**DOI:** 10.1186/1939-4551-8-S1-A232

**Published:** 2015-04-08

**Authors:** Carlos E Baena-Cagnani, Ashok Mahashur, Jamal Jawad, Margarita Murrieta-Aguttes, Faheem A Tadros, Mohammad Gharagozlou, Talha Mahmud

**Affiliations:** 1 Research Centre in Respiratory Medicine, Argentina; 2 Hinduja Hospital, India; 3 Dr Soliman Fakeeh Hospital, Saudi Arabia; 4 Sanofi, France; 5 Al Zahra Hospital, United Arab Emirates; 6 Children's Medical Center, Iran; 7 Pakistan

## Background

Allergic Rhinitis (AR) is a worldwide spread illness having an important impact on social life, sleep quality, school, and work productivity and huge costs. Patient preference is becoming an important aspect in medical care. ISMAR was designed as the first-over global survey to identify differences in attitudes and preference in patients and physicians about AR. ISMAR 2 is the second phase.

## Methods

ISMAR is an international, multicenter, non-interventional, cross-sectional study in adults and children (≥ 6 years) with physician-diagnosed AR of at least 1 year of duration conducted in Algeria, Egypt, United Arab Emirates, Qatar, Kuwait, India, Islamic Republic of Iran, Pakistan, and Saudi Arabia.

Physicians (GPs/Family doctors/internists, pediatricians, allergists/pulmonologists, and ENT) working in public and private sectors or both were selected from master lists of physicians attending AR patients and invited to participate in the study. They answered to the Investigator’s Questionnaire (guidelines awareness, relevant AR symptoms, and preference for prescribing medication among others) and recruited consecutive patients to whom the ISMAR questionnaire was administered. Data collection was performed during a single visit. A patient's questionnaire and a Case Record Form were also filled in. Statistical analysis was descriptive. Herein we are showing the physician's view.

## Results

One hundred and seventy eight physicians participated. They were aware about ARIA (82.6%) and GINA (76.4%) and followed them to classify patients severity (83.7%) and for choosing the treatment accordingly (82.0%). Key symptoms to diagnose AR were congestion (85.4%), sneezing (93.3%), and anterior watery rhinorrhea (89.3%). SQ and AR severity were assessed mainly by clinical history (93.8% and 98.7 %). The most commonly tests were serum total IgE (52.8%), eosinophilia (50.0%), x-rays (48.9%), CT scan (44.4%), and allergen skin tests (39.3%). The main reasons to prescribe medication were symptom severity/frequency (98.3 %), drug efficacy (80.3% %) and safety (78.7 %). Other less relevant reasons were personal experience (60.7 %), route of administration (55.1%), cost (52.8%), and frequency of doses (48.9%). The preferred medications were oral anti-H_1_ antihistamines (Oa-H_1_: 68.5%) and intranasal corticosteroids (ICS: 64.0 %) [5 in a 0–5 scale].

## Conclusions

Guidelines are well known and useful to physicians. Clinical history was the main instrument to evaluate patient's SQ, classification and severity of AR, and choice of treatment. Allergen skin tests were not commonly performed. Oa-H_1_ and ICS were the most widely recommended treatment for AR and were considered effective and safe.

